# Frequency, clinical presentation and outcome of vigilance impairment in patients with uni- and bilateral ischemic infarction of the paramedian thalamus

**DOI:** 10.1007/s00415-021-10565-y

**Published:** 2021-04-21

**Authors:** Merve Fritsch, Kersten Villringer, Ramanan Ganeshan, Ida Rangus, Christian H. Nolte

**Affiliations:** 1 Department of Neurology, Charité Universitätsmedizin Berlin, corporate member of Freie Universität Berlin, Humboldt-Universität zu Berlin and Berlin Institute of Health, Charitéplatz 1, 10117 Berlin, Germany; 2 Center for Stroke Research Berlin, Charité Universitätsmedizin Berlin, corporate member of Freie Universität Berlin, Humboldt-Universität zu Berlin and Berlin Institute of Health, Berlin, Germany; 3 Department of Psychiatry and Psychotherapy, CCM, Charité-Universitätsmedizin Berlin, corporate member of Freie Universität Berlin, Humboldt-Universität zu Berlin and Berlin Institute of Health, Berlin, Germany

**Keywords:** Vigilance impairment, Ischemic stroke, Paramedian thalamus, Outcome

## Abstract

Ischemic stroke of the paramedian thalamus is a rare differential diagnosis in sudden altered vigilance states. While efforts to describe clinical symptomatology exist, data on the frequency of paramedian thalamic stroke as a cause of sudden impaired vigilance and on accompanying clinical signs and outcome are scarce. We retrospectively analyzed consecutive patients admitted to a tertiary stroke center between 2010 and 2019 diagnosed with paramedian thalamic stroke. We evaluated frequency of vigilance impairment (VI) due to paramedian thalamic stroke, accompanying clinical signs and short-term outcome in uni- versus bilateral paramedian lesion location. Of 3896 ischemic stroke patients, 53 showed a paramedian thalamic stroke location (1.4%). VI was seen in 29/53 patients with paramedian thalamic stroke and in 414/3896 with any stroke (10.6%). Paramedian thalamic stroke was identified as causal to VI in 3.4% of all patients with initial VI in the emergency department and in 0.7% of all ischemic stroke patients treated in our center. Accompanying clinical signs were detected in 21 of these 29 patients (72.4%) and facilitated a timely diagnosis. VI was significantly more common after bilateral than unilateral lesions (92.0% vs. 21.4%; *p* < 0.001). Patients with bilateral paramedian lesions were more severely affected, had longer hospital stays and more frequently required in-patient rehabilitation. Paramedian thalamic lesions account for about 1 in 15 stroke patients presenting with impaired vigilance. Bilateral paramedian lesion location is associated with worse stroke severity and short-term outcome. Paying attention to accompanying clinical signs is of importance as they may facilitate a timely diagnosis.

## Introduction

Sudden impaired vigilance is a common diagnostic challenge due to numerous differential diagnoses. Seldom, it may be caused by ischemic stroke in the paramedian thalamus [1]. The paramedian thalamus is typically supplied by the paramedian artery (also called mesencephalic artery), arising from the P1-segment of the posterior cerebral artery (PCA). The occlusion of the “Artery of Percheron”, an anatomical variation whereby both paramedian arteries arise from a common P1, often leads to bilateral paramedian thalamic infarction [2]. Paramedian lesions account for 25% of all thalamic but only for 0.6% of all ischemic strokes, indicating it to be a rare stroke lesion location [3]. Their seldom occurrence and heterogeneity of clinical presentation might lead to a delay or even misclassification of diagnosis [4]. Since paramedian lesions have been described to result in vigilance impairment to the extent of comatose states, knowledge on frequency, clinical presentation and of prognosis may help to facilitate timely diagnoses and guide further therapeutic measures [5]. In addition to vigilance impairment, clinical presentation may include gaze palsy and sensorimotor symptoms. Identification of these may help the clinician to the correct diagnosis [6]. However, data on frequency of paramedian thalamic stroke as cause of vigilance impairment, frequency of accompanying clinical signs and outcome of uni- and bilateral paramedian thalamic lesions are scarce.

### Aim

We investigated the frequency, clinical presentation and short-term outcome of ischemic paramedian thalamic lesions in a large cohort of ischemic stroke patients to further understand the role of the paramedian thalamus in sudden vigilance impairment. We additionally analyzed what factors might influence facilitation of a timely diagnosis.

## Methods

The data that support the findings of this study are available from the corresponding author upon reasonable request.

### Participants

We conducted a retrospective analysis of consecutive stroke patients who were admitted to the Stroke Unit or Intensive Care Unit of the Charité Campus Benjamin Franklin in Berlin between 2011 and 2019. We screened for the frequency of vigilance impairment as a presenting symptom and for the frequency of thalamic strokes. We then analyzed the lesion location in patients that had received an MRI showing a lesion confined to the thalamus. For lesion analysis, patients were categorized into uni- and bilateral paramedian thalamic lesions.

In addition, we calculated the rate of uni- and bilateral paramedian thalamic lesions among all neurologic cases presenting with “sudden vigilance impairment” of unknown cause admitted to our emergency department (ED) during the observation period. The latter diagnosis was given after exclusion of obvious causes such as metabolic (e.g., hypoglycemia) or circulatory disease (e.g., syncope), intracranial disease (e.g., intracranial bleeding, large hemispheric ischemic stroke, large vessel occlusion), infectious disease and seizures.

### Assessment of vigilance and prognosis

Patients’ vigilance states were assessed by board-certified physicians according to item 1A of the National Institutes of Health Stroke Scale (NIHSS; Level of consciousness, 0–3 points) which corresponds to the “eye opening” part of the Glasgow Coma Scale (GCS) [7, 8]. Patients received standardized vigilance testing at least every 6 h for the duration of their stay on the stroke unit (minimum of 24 h). If patients scored 1 or more points on this item, vigilance impairment was assumed. The number of days in which patients showed vigilance impairment and the number of days under stroke unit supervision were analyzed (as in 24-h units).

Outcome was assessed by comparing patients’ stroke severity (NIHSS) and degree of independence (mRS) upon admission and discharge, their need for in- or outpatient rehabilitation as well as in-hospital death as a consequence of stroke. In addition, the impact of paramedian stroke location on outcome was investigated by comparing degree of independence (as defined by mRS) at discharge between paramedian and other stroke locations.

### Assessment of manifestation of other clinical symptoms

The presence of gaze palsy as well as motor or sensory deficits were analyzed according to item 2 of the NIHSS (Horizontal Eye Movement, 0–2 points) and the corresponding items for motor (items 5 and 6) and sensory function (item 9) on the NIHSS. Stroke etiology was categorized according to the discretion of the treating physician and followed the TOAST criteria [9]. Of note, stenosis of > 50% in the supplying artery was not mandatory for categorization as “large artery atherosclerosis”. Instead, any atherosclerosis was considered.

### Imaging methods

MRI is readily available on the stroke unit and routinely administered to all patients with suspected or proven stroke [10]. Exceptions apply to patients with contraindication of MRI (i.e., implanted electronical devices, high risk of undergoing MRI, i.e., due to unstable vital signs) and patients not willing to undergo MRI in absence of an emergency. These patients receive CCT-imaging. CCT-imaging in routine clinical care was performed using a Siemens Somatom scanner (Siemens Medical Solutions, Erlangen, Germany). MRI was performed in routine clinical care using a 3 T Siemens Magnetom Trio scanner (Siemens Medical Solutions, Erlangen, Germany). The MRI imaging protocol consisted of T2*, fluid attenuation inversion recovery (FLAIR), diffusion weighted imaging (DWI, slice thickness 2.5 mm) and MR time-of-flight angiography (MR-TOF). Images were analyzed by board-certified neuroradiologists. Anatomical lesion location within the thalamus was determined based on a study by Carrera/Bogousslavsky in which the thalamus was divided into four anatomical areas (anterior, paramedian, inferolateral, and posterior) [11].

### Statistical analysis

Statistical analysis was performed using SPSS 24.0 (IBM, Armonk, NY) and MATLAB 2017b (The MathWorks, Natick, MA). Association between vigilance impairment and lesion location as well as nominal data was evaluated using Chi-Square test, due to the small sample size. Group comparison for number of days in care/with vigilance impairment and age was evaluated using Mann–Whitney *U* Test for independent samples with non-parametric distribution. All statistical tests were carried out with a significance level at α = 0.05.

## Results

We analyzed 3896 consecutive stroke patients being admitted to either the Stroke Unit or Intensive Care Unit of the Charité Campus Benjamin Franklin. We additionally analyzed 59,710 consecutive neurologic patients presenting to the emergency department (ED) of the same hospital.

### Frequency of vigilance impairment and thalamic stroke

Out of 3896 ischemic stroke patients being admitted, 414 showed symptoms of vigilance impairment upon admission (10.6%), 29 of which were classified as having an isolated lesion in the thalamus. Of these, all were in paramedian location (29/414, 7.0% of vigilance impaired stroke patients and 0.7% of all stroke patients respectively). A stroke confined to any location of the thalamus was seen in 146/3896 patients (3.7% of all stroke patients). A uni- or bilateral ischemic stroke that was confined to the paramedian thalamus was seen in 53 patients (1.4% of all stroke patients, 25 bilateral, 28 unilateral). Of the unilateral lesions, 16 were left- and 12 right-sided paramedian lesions. While all patients showing vigilance impairment after a thalamic lesion had a paramedian lesion location, only 29/53 patients with a paramedian thalamic lesion location showed vigilance impairment due to stroke (54.7%).

**Fig. 1:**
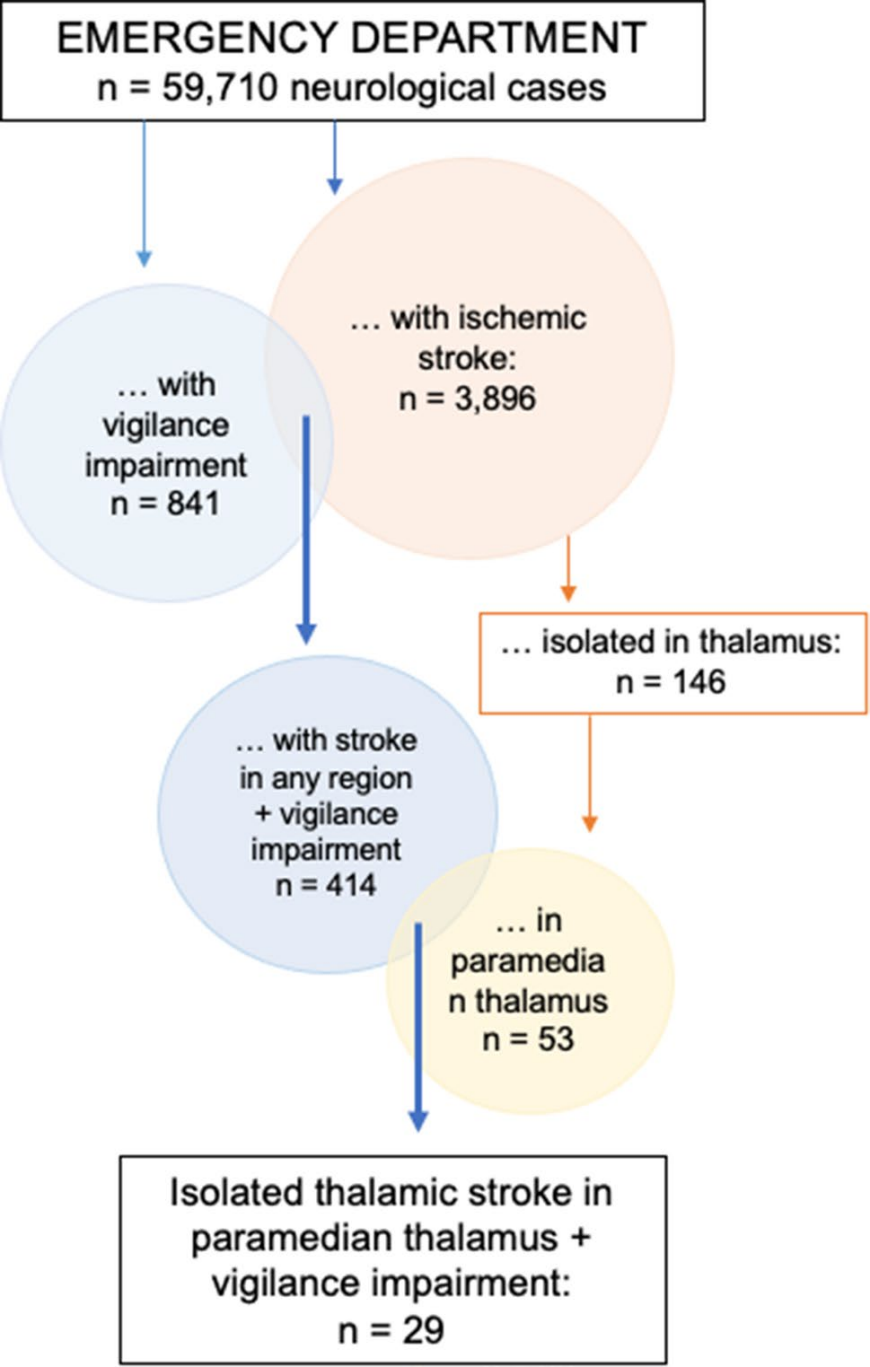
Flowchart characterizing recruitment of patients as well as frequency of ischemic stroke of the paramedian thalamus and of vigilance impairment following stroke

Out of 59,710 consecutive neurologic patients presenting to the ED, 841 presented with vigilance impairment (1.4%), 29 of which were identified with a paramedian thalamic stroke (3.5%). Further detailed analyses are based on the 53 patients with paramedian thalamic stroke (overview Fig. 1). Patients with uni- and bilateral paramedian lesions as well as patients with and without vigilance impairment did not differ with respect to age, sex, cardio-vascular risk factors or presumed etiology of stroke (Tables 1, 2).

**Table 1:** Demographics, clinical presentation and outcome in patients with uni- and bilateral thalamic strokes (*n* = 53)

	Unilateral (*n* = 28)	Bilateral (*n* = 25)	*p*^a^
Demographics			
Age (years)	69.44 ± 15.30	73.38 ± 16.38	0.85
Sex (female)	57.1%	44%	0.35
Diabetes	13.0%	27.3%	0.25
Hypertension	47.8%	84.6%	0.13
Atrial fibrillation	17.2%	28.4%	0.38
Presumed etiology of stroke			
Large-vessel atherosclerosis	28.6%	24.0%	0.13
Cardioembolic	17.9%	20.0%	0.78
Small-vessel atherosclerosis	32.1%	20.0%	0.53
Other	0%	12.0%	0.09
Concurring etiologies	10.7%	12.0%	0.25
Cryptogenic	10.7%	12.0%	0.28
Severity of stroke			
Vigilance impairment admission	21.4% (6/28)	92.0% (23/25)	* < 0.001
Duration of vigilance impairment	0.5 ± 1.5 days	1.9 ± 3.3 days	*0.005
Stroke severity on admission (NIHSS)^b^	4 (median)	11 (median)	* < 0.001
Degree of dependence on admission (mRS)^c^	2 (median)	4 (median)	* < 0.001
Prognosis and accompanying clinical signs			
Degree of dependence improvement (mRS, admission to discharge)	1 (median)	2 (median)	*0.04
Stroke severity improvement (NIHSS, admission to discharge)	2 (median)	1 (median)	*0.02
In-hospital death	0% (0/28)	28% (7/25)	*0.003
Rehabilitation (any)	39.3% (11/28)	94.4% (17/18)	*0.001
Rehabilitation (in-patient)	25.0% (7/28)	83.3% (15/18)	*0.001
Duration of hospital stay	3 ± 1.7 days	5 ± 3.4 days	*0.006
Additional gaze palsy	32.1% (9/28)	58.3% (14/24)	0.176
Additional sensory motor symptoms	39.3% (11/28)	52.0% (13/25)	0.415

*Significant results, significance level of *p* < 0.05

^a^Mann–Whitney *U* Test continuous variables, Chi-Square test for categorical variables

^b^NIHSS: National Institutes of Health Stroke Scale

^c^mRS: Modified Ranking Scale

**Table 2:** Differences between patients with and without vigilance impairment (*n* = 53)

	Vigilance impairment (VI, *n* = 29)	No vigilance impairment (non-VI, *n* = 24)	*p*^a^
Demographics			
Age (years)	71.92 ± 17.17	70.35 ± 14.35	0.73
Sex (female)	51.7%	50%	0.56
Diabetes	26.9%	10.5%	0.26
Hypertension	65.4%	52.6%	0.54
Atrial fibrillation	21.9%	16.0%	0.63
Bilateral location	79.3% (23/29)	8.3% (2/24)	* < 0.001
Prognosis, accompanying symptoms			
In-hospital death	24.1% (7/29)	0% (0/24)	*0.012
Rehabilitation (any)	90.9% (20/22)	33.3% (8/24)	*< 0.001
Rehabilitation (in-patient)	53.8% (7/13)	22.2% (4/18)	*0.05
Stroke severity admission (NIHSS)^b^	10 (median)	3 (median)	*< 0.001
Stroke severity improvement (NIHSS, admission to discharge)	2 (median)	1 (median)	*< 0.001
Degree of dependence on admission (mRS)^c^	4 (median)	2 (median)	*< 0.001
Degree of dependence on improvement (mRS, admission to discharge)	1 (median)	1 (median)	0.06
Duration of hospital stay	5.8 ± 2.6 days	3.4 ± 1.5 days	*0.018
Gaze palsy	62.1% (18/29)	25.0% (6/24)	*0.013
Sensory motor symptoms	48.3% (14/29)	41.7% (10/24)	0.96
Intravenous thrombolysis	20.6% (6/29)	12.5% (3/24)	0.23

*Significant results, significance level of *p* < 0.05

^a^Mann–Whitney *U* Test continuous variables, Chi-Square test for categorical variables

^b^NIHSS: National Institutes of Health Stroke Scale

^c^mRS: Modified Ranking Scale

### Severity of vigilance impairment, clinical impairment and outcome in uni- vs. bilateral thalamic lesions

Vigilance impairment upon admission was reported significantly more often in patients with bilateral paramedian infarction than in unilateral paramedian infarction (unilateral: 6/28, 21.4% vs. bilateral 23/25, 92.0%, *p* < 0.001) (Table 1). Severity of vigilance impairment was more pronounced in patients with bilateral infarctions than in unilaterally affected patients. All patients with bilateral infarctions scored at least 2 points on item 1a of the NIHSS (“Requires repeated stimulation to arouse”) while 85% of patients with a unilateral infarction scored 0 points (“alert”) (bilateral: median 2; unilateral: median 0 (range 0–3); *p* = 0.001). In addition, patients with bilateral infarctions scored an average of 2 points on both items 1b and 1c (“no question answered correctly”, “no task performed correctly”).

The average duration of vigilance impairment was significantly longer in patients with bilateral than unilateral stroke manifestation (mean days of vigilance impairment, bilateral: 1.9 ± 3.3, unilateral: 0.5 ± 1.5; *p* = 0.005), with one patient showing mild vigilance impairment for up to 6 days after a unilateral, paramedian infarction and one patient showing a severe, comatose state for 11 days following bilateral paramedian infarction (Table 1).

**Fig. 2:**
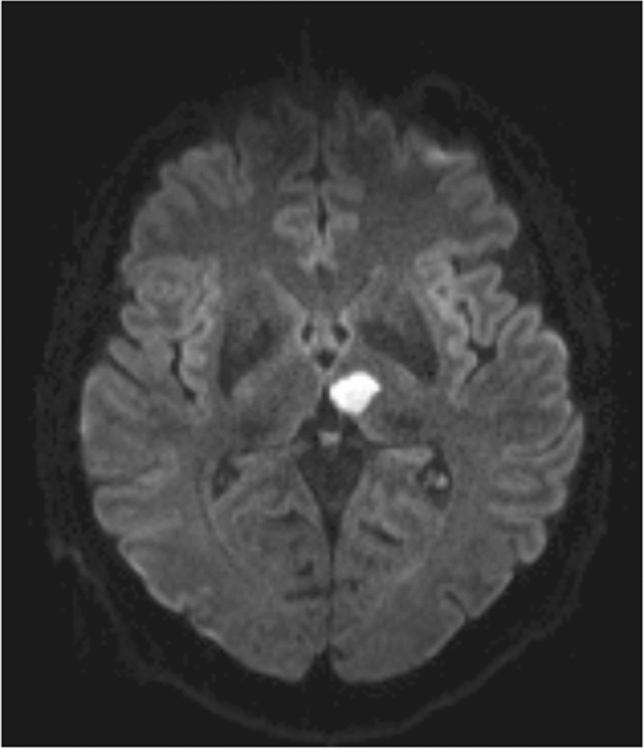
Example of a unilateral, paramedian ischemic lesion in the left thalamus (DWI imaging)

**Fig. 3:**
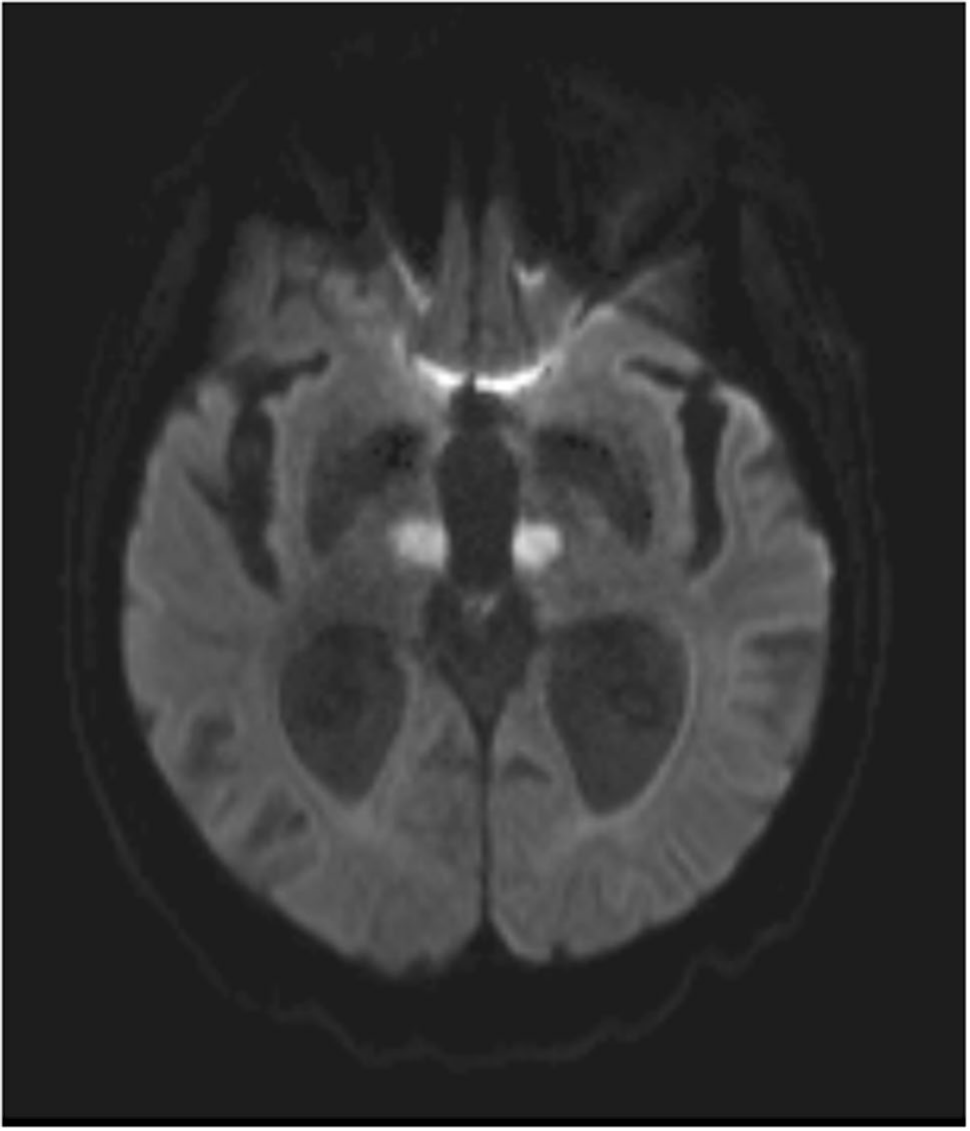
Example of bilateral, paramedian ischemic lesion in the thalamus (DWI imaging)

When comparing overall clinical severity, patients with bilateral paramedian thalamic infarction presented with significantly higher scores in both the NIHSS as well as the mRS upon admission, than patients with a unilateral lesion (median NIHSS, bilateral: 11 points, unilateral: 4 points (range: 2–11); p < 0.001; median mRS, bilateral: 4 points, unilateral: 2 points (range 2–4); *p* < 0.001). Patients with bilateral lesions also showed significantly poorer improvement of overall symptom severity (median NIHSS improvement, unilateral: 2 points, bilateral: 1 point, *p* = 0.02). The average length of hospital stay on the stroke unit was significantly longer in patients with bilateral paramedian infarctions than in unilateral lesion location (mean days duration of hospital stay, bilateral: 5 ± 3.4, unilateral: 3 ± 1.7; *p* = 0.006). Seven of 24 patients with a bilateral lesion died, while none of the unilaterally affected patients did (bilateral: 7/24, 28%, unilateral: 0/28, 0%, *p* = 0.003). Surviving patients with bilateral paramedian lesions also showed a significantly higher need for further in- or outpatient rehabilitative treatment (unilateral: 11/28, 39.3%; bilateral: 17/18, 94.4%, *p* = 0.001), with 83.3% of bilaterally affected patients and 25% of unilaterally affected patients needing in-patient rehabilitation (*p* = 0.001) (Table 1, Figs. 2, 3).

### Severity of clinical impairment and outcome of patients with and without vigilance impairment

When comparing thalamic stroke patients with and without vigilance impairment upon admission, patients with vigilance impairment (VI) showed significantly higher scores in NIHSS and mRS, than patients without vigilance impairment (non-VI) (median NIHSS, VI: 10 points, non-VI: 3 points (range 0–18), *p* < 0.001; median mRS, VI: 4, non-VI: 2 (range 0–4), *p* < 0.001). Approximately, 1 in 3 patients (9/29) with vigilance impairment had to be treated on the intensive care unit (ICU). Seven of the 29 patients with initial vigilance impairment (24.1%), but none of the patients who were initially awake, died (*p* = 0.012). In the surviving 22 patients, who were initially vigilance impaired, overall NIHSS improved by a mean of two points and mRS improved by a mean of one point. None of the surviving patients showed signs of vigilance impairment upon discharge. All 22 patients were discharged awake. The vast majority (20/22, 90.9%) of the surviving patients with initial vigilance impairment, while only a third of the non-VI patients received further in- or outpatient rehabilitative treatment (Table 2).

Paramedian thalamic stroke location did not impact on the degree of independence at discharge (median mRS discharge, paramedian thalamic strokes: 2, other stroke locations: 2, range: 0–6, Wilcoxon Rank Sum *p* = 0.38). Adjusting for stroke severity, age, intravenous thrombolysis and co-morbidities did not alter lack of association (multinomial logistic regression for ordinal responses: *T*(9257) = 1.02, *p* = 0.76).

### Additional symptoms in patients with thalamic lesions with and without vigilance impairment

Out of all 53 patients with thalamic stroke, 23 (43.4%) presented with additional gaze palsy and 24 (54.3%) presented with additional sensorimotor symptoms upon admission.

Gaze palsy was overall more likely in patients with vigilance impairment (VI: 18/29, 62.1%; non-VI: 6/24, 25%, *p* = 0.013). The presence of gaze palsy did not differ significantly between patients with a uni- or bilateral lesion and vigilance impairment (unilateral: 3/6 (50%); bilateral: 15/23 (65.2%), *p* = 0.41).

Sensorimotor symptoms were not more common in patients with vigilance impairment (VI: 14/29, 48.3%; non-VI: 10/24, 41.7%, *p* = 0.964) and also did not differ significantly between patients with uni- or bilateral lesion location and vigilance impairment (VI unilateral: 4/6, 33.3%, VI bilateral: 12/23, 65.5%, *p* = 0.51). Overall, 21/29 patients with vigilance impairment and paramedian thalamic stroke presented with either gaze palsy or sensorimotor symptoms (72.4%) (Table 2).

### Time to diagnosis in vigilance impaired patients with thalamic stroke (*n* = 29)

Mean time to diagnosis (TtD) of the 29 patients with initially impaired vigilance and thalamic lesions after admission to the ED or Stroke Unit was 16.4 h. Time to diagnosis was shorter (i.e., diagnosis was made faster) in patients that showed additional horizontal or vertical gaze palsy (mean TtD, with gaze palsy: 3.8 ± 6.5 h, without gaze palsy: 36.9 ± 39.1 h; *p* = 0.019) or additional sensorimotor symptoms (mean TtD, with sensorimotor symptoms: 5.3 ± 12.6 h, without sensorimotor symptoms: 32.1 ± 3 7.9 h; *p* = 0.035).

## Discussion

The aim of our study was to identify the frequency of paramedian thalamic infarction as a cause of sudden vigilance impairment and to analyze the occurrence of accompanying clinical symptoms. In addition, we wanted to delineate prognosis and short-term outcome.

In our cohort of 3896 acute stroke patients, about 10% showed vigilance. However, vigilance impairment due to thalamic stroke was rare (~ 0.7%) and confined to a paramedian thalamic stroke location. Bilateral paramedian strokes entailed loss of consciousness more often than unilateral lesions. Additional clinical signs, such as gaze palsy, were only seen in about half of thalamic stroke patients with vigilance impairment, but can nevertheless facilitate a timely diagnosis. Outcome of patients was less favorable in bilateral paramedian strokes.

### Frequency of vigilance impairment in thalamic strokes

Due to the large number of possible differential diagnoses, vigilance impairment poses a common diagnostic challenge. In our large cohort of ischemic stroke patients, paramedian thalamic infarction was the cause of sudden altered vigilance impairment in about 7% of ischemic stroke patients and 3.5% of patients presented to an ER due to vigilance impairment. Generally, ischemic stroke mediates vigilance impairment by size causing increased intracranial pressure and brain shift as seen in malignant middle cerebral artery (MCA) or, alternatively, by large brainstem involvement [12, 13]. In these cases, clinical presentation tends to be more typical of a stroke, including hemiparesis or oculomotor symptoms, prompting fast neurologic assessment and therapy. Thalamic lesions, on the other hand, are often missed, as typical clinical signs are subtle or altogether absent [4]. Indeed, gaze palsy and sensorimotor symptoms were absent in 19 of our cohort of 53 paramedian thalamic stroke patients, which makes paramedian thalamic stroke a challenging diagnosis. While overall frequency of paramedian, thalamic ischemic lesions is small, its relevance in sudden vigilance impairment has none the less been stressed [3].

### Differences in frequency, severity and outcome of vigilance impairment in uni- vs. bilateral paramedian lesions

While case reports had previously pointed towards a role of specifically bilateral paramedian thalamic lesions in loss of consciousness, altered vigilance states can also occur following unilateral paramedian thalamic lesions as well as after ischemia in other thalamic areas [1, 5, 14].

Superior outcome has been indicated in initially vigilance impaired, bi-thalamic stroke patients without involvement of the rostral midbrain [5]. In addition, vigilance impairment has been suggested to be slightly more frequent in bilateral, paramedian lesions [14]. However, previous studies provided only small sample sizes and often either included patients with additional lesions or did not take unilateral lesions into account. In our cohort, vigilance impairment after thalamic stroke exclusively followed lesions in the uni- or bilateral paramedian thalamus, with vigilance impairment upon admission being three times more common in bilateral paramedian lesions.

Patients also differed in clinical presentation as well as certain outcome measures. Vigilance impairment and overall clinical affectedness was more severe in patients with bilateral lesions. About one in three patients with vigilance impairment due to paramedian thalamic stroke had to be treated on an ICU, all of which were affected bilaterally. In addition, duration of vigilance impairment was three times longer with consecutive longer hospital stays and patients with bilateral lesions remaining more severely affected after discharge which resulted in a higher rate of in- or outpatient rehabilitation. About a quarter of patients with a bilateral paramedian lesion location died, while none of the patients affected unilaterally succumbed to their stroke.

Similar results were reported by Bogousslavsky et al. and Zappella et al., who found comatose states in both uni- as well as bi-paramedian ischemic lesions but observed a more rapid improvement in unilaterally affected patients only [15, 16]. In addition, Weidauer et al. saw persistent vigilance impairment in follow-up consultation in 50% of patients with bilateral paramedian infarction in their cohort [17]. We could not show a specific impact of paramedian thalamic lesion location on functional outcome at discharge, when comparing with other stroke lesion locations. This may have been due to the small number of patients with this stroke location. However, it should reassure the optimistic therapeutical approach in these patients.

While many accounts have been made to describe the role of the thalamus in vigilance, it is not fully understood why specifically paramedian location is important and bilateral lesions lead to a higher severity.

As the receiving organ of the ascending reticular activating system (ARAS), thalamic nuclei are thought to control sleep–wake-transitions [18, 19]. In addition, a shift towards tonic activity of thalamic nuclei is needed for the transfer of sensory information to the cortex [20]. Specifically, stimulation studies in mice have shown that the paraventricular thalamic nucleus (PVT), located in the paramedian thalamus, is a main driver of circadian rhythm, thereby stressing its importance in wakefulness [21, 21]. This could be explanatory of our result of mainly paramedian, thalamic lesions leading to vigilance impairment. Furthermore, other bilateral stroke syndromes are also known to lead to more severe and prolonged symptomatology, such as cortical blindness due to bi-occipital lesions, acute mutism due to bilateral caudate head infarction and locked-in-syndrome due to a top-of-the-basilary stroke. Hence, similar accounts could be made for our result, whereby bilateral paramedian lesions lead to a more severe symptomatology and outcome [23–25].

### Frequency of additional symptoms and their effect on time to diagnosis as well as outcome

In our sample, only 60% of patients with paramedian lesions and vigilance impairment showed additional gaze palsy. Moreover, only about half of these patients showed additional sensorimotor symptoms. Gaze palsy and other forms of oculomotor symptoms are known to occur in infarction of the artery of Percheron and after isolated thalamic or complex midbrain infarctions. However, frequency of presentation in isolated lesions of the paramedian thalamus and co-occurrence with vigilance impairment are not known [24, 26]. Previous studies have shown the occurrence of vertical gaze palsy or complex oculomotor symptoms in up to 40% of patients with uni- or bilateral paramedian strokes. However, patients with additional midbrain lesions were not excluded, making correlation to specifically thalamic infarction difficult [14]. Furthermore, no account was made for the co-occurrence of altered vigilance states [27]. Our results, showing a significant association of presence of gaze palsy with vigilance impairment in up to 60% of patients, stress the importance of screening for accompanying oculomotor symptoms.

Of note, time to diagnosis (TtD) was significantly shorter when patients presented with additional symptoms such as gaze palsy or sensorimotor symptoms. Patients, who presented with such accompanying symptoms were diagnosed with an ischemic stroke almost 30 h earlier than patients, that only showed vigilance impairment without accompanying deficits. As this could impact further treatment options, it stresses the importance of thoroughly screening for accompanying symptoms, especially suggesting midbrain lesions, when confronted with sudden altered vigilance impairment of unknown cause in the emergency setting.

### Limitations

Limitations of this analysis include selection bias due to its monocentric and retrospective design. Second, the number of patients with paramedian thalamic stroke and/or sudden onset vigilance impairment might be underestimated because not all patients underwent MRI. Rate of MRI is very high in our stroke center, as MRI is routinely administered to almost all patients, especially when presenting with unspecific syndromes such as sudden onset vigilance impairment. However, MRI is not feasible in every patient. Therefore, we cannot provide a number for the amount of patients with vigilance impairment due to thalamic stroke that have slipped our diagnostic approach, as some patients might have received CCT only due to MRI-contraindications. Third, some patients with transient vigilance impairment might have fully recovered in the pre-hospital phase, excluding them from our sample. Fourth, while we were able to provide “Time to Diagnosis” as a surrogate marker for delayed or missed diagnoses in patients with thalamic stroke with and without vigilance impairment, we were not able to compare this parameter for all patients of the sample, as it is not routinely collected. Finally, we cannot provide long-term follow-up due to the retrospective design of the study.

## Summary

We were able to show that ischemic stroke of the paramedian thalamus is a rare cause of sudden vigilance impairment. Vigilance impairment is more common and more pronounced in bilateral than unilateral thalamic lesions. Bilateral paramedian lesions show more severe neurological deficits on admission and poorer short-term outcomes. Only about half of patients with paramedian thalamic lesions due to ischemic stroke show additional clinical signs, such as gaze palsy or sensory motor symptoms upon admission but the presence of such symptoms facilitates faster diagnosis.

## Data Availability

The data that support the findings of this study are available from the corresponding author upon reasonable request.
